# On-Line Preconcentration of Selected Kynurenine Pathway Metabolites and Amino Acids in Urine via Pressure-Assisted Electrokinetic Injection in a Mixed Micelle System

**DOI:** 10.3390/ijms26136125

**Published:** 2025-06-26

**Authors:** Michał Pieckowski, Ilona Olędzka, Tomasz Bączek, Piotr Kowalski

**Affiliations:** 1 Department of Pharmaceutical Chemistry, Medical University of Gdańsk, Hallera 107, 80-416 Gdańsk, Poland; mpiec@gumed.edu.pl (M.P.); ilona@gumed.edu.pl (I.O.); tbaczek@gumed.edu.pl (T.B.); 2 Department of Nursing and Medical Rescue, Institute of Health Sciences, Pomeranian University in Słupsk, 76-200 Słupsk, Poland

**Keywords:** capillary electrokinetic chromatography, pressure-assisted electrokinetic injection, mixed micelle, anionic compounds, kynurenine pathway, on-line preconcentration

## Abstract

To enhance the signal intensity of kynurenines, which are present at trace concentrations in biological fluids, a novel analytical approach was developed, combining pressure-assisted electrokinetic injection (PAEKI) with a mixed micelle system based on sodium dodecyl sulfate (SDS) and Brij-35. The method was applied to key compounds of the kynurenine pathway, including L-tryptophan, kynurenine, 3-hydroxykynurenine, and kynurenic acid, as well as to the aromatic amino acids (AAs) L-tyrosine and L-phenylalanine. PAEKI was performed by electrokinetic injection for 2 min at −6.5 kV (reversed polarity) and 0.5 psi (3.45 kPa) using a fused silica capillary (50 cm in length, 50 µm inner diameter). The background electrolyte (BGE) consisted of 20 mM Na_2_B_4_O_7_ (pH 9.2), 2 mM Brij-35, 20 mM SDS, and 20% (*v*/*v*) methanol (MeOH). The limit of detection (LOD) using a diode array detector (DAD) was 1.2 ng/mL for kynurenine and ranged from 1.5 to 3.0 ng/mL for the other analytes. The application of PAEKI in conjunction with micellar electrokinetic capillary chromatography (MEKC) and solid-phase extraction (SPE) of artificial urine samples resulted in a 146-fold increase in signal intensity for kynurenines compared to that observed using the hydrodynamic injection (HDI) mode. The developed method demonstrates strong potential for determining kynurenine pathway metabolites in complex biological matrices.

## 1. Introduction

**Figure 1:**
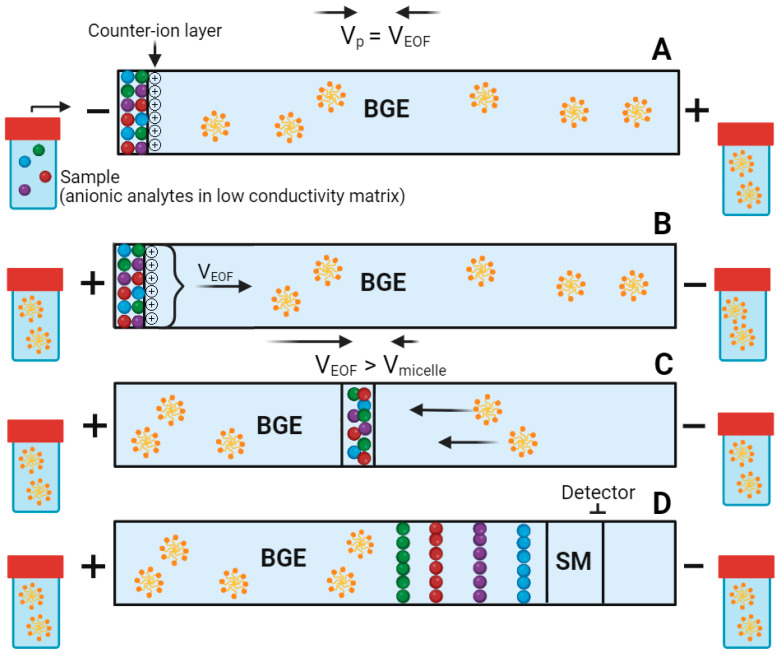
(**A**) In the initial situation, a sample with low conductivity (0.01 mM NaOH) is present at the inlet of the capillary, while a micellar BGE is located at the outlet. Voltage is applied with reversed electrode polarity, simultaneously utilizing pressure that counterbalances the EOF, which is directed opposite to the applied pressure. The analytes preconcentrate at the sample/BGE interface, where a layer of counterions (cations) stabilizes it. (**B**,**C**) The sample at the capillary inlet is replaced by the BGE, and the electrode polarity is switched to normal, leading to the migration of accumulated anions. In this case, the EOF is directed toward the capillary outlet (cathode), and its value exceeds the migration velocities of the micelles. Mixed micelles of SDS and Brij-35, which have a net negative charge and an apparent migration direction toward the anode (note that *V_EOF_* > *V_micelle_*), interact with the analytes, enhancing the separation potential. (**D**) The separated and stacked analytes migrate through the detection window. SM—sample matrix.

PAEKI utilizes samples containing ionized analytes with ionic strength significantly lower than that of the BGE (Figure 1). This configuration balances the electroosmotic flow (EOF) velocity against an external hydrodynamic pressure, resulting in a stationary boundary at the capillary inlet where analytes undergo stacking [1]. Zhang et al. [2] described the mechanism of PAEKI; the amount of analytes injected over time *t*, denoted as *N_i_*(*t*), can be described by the equation:

$$N_{i}\left(t\right)=\gamma \mu_{ep}E_{inj}AC_{inj}t$$

The factor γ accounts for analyte accumulation effects caused by the counter-ion layer, which limits diffusion during PAEKI. γ is defined as the ratio of the resistivity of the sample matrix to that of the BGE, and as such, it is a dimensionless parameter. In the original equation, Zhang et al. used the notation “$\mu_{ep,A}$”, which represents the electrophoretic mobility of the analyte in the sample solution; this was changed for $\mu_{ep}$. “*A*” is the cross-sectional area of the capillary, $E_{inj}$ is the electric field, $C_{inj}$ denotes the concentration of the analyte in the sample solution, and *t* is the injection time. In accordance with IUPAC recommendations, the unit on the right-hand side of the equation is the mole (mol):

$$\frac{m^{2}}{V\times s}\times \frac{V}{m}\times m^{2}\times \frac{mol}{m^{3}}\times s=mol$$

PAEKI employs the applied pressure to counteract the reversed EOF within the capillary during sample injection, while simultaneously leveraging the enhanced electric field in the sample zone. In equilibrium, the BGE in the capillary remains stationary, theoretically allowing for unlimited injection time and potentially unlimited peak enhancement. PAEKI generates a narrow sample zone within the capillary by forming a phase boundary, which leads to a highly concentrated band of ionized analytes due to ion retardation at the interface with the BGE [2].

PAEKI has been successfully applied for the preconcentration of mononucleotides, oligonucleotides, monophthalates, and halogenated phenols [1,2,3,4,5]. The most common application of PAEKI is in combination with capillary zone electrophoresis (CZE), where it has been used to analyse sulphonamides [6], biogenic amines [7], phenols [8], and haloacetic acids [9]. Additionally, a modification of the BGE with methyl-β-cyclodextrin has been employed for the separation of fluoroquinolones [10]. Sodium cholate was also used to modify the BGE for the separation of verteporfin enantiomers [11]. Recently, Pieckowski et al. [12] were the first to apply PAEKI to hydrophobic compounds (fat-soluble vitamins) in nanoemulsion matrices, in combination with microemulsion electrokinetic chromatography (MEEKC). In a recent study, our team developed a novel method for the determination of fluorescent derivatives of thyroid hormones in urine samples using PAEKI for signal enhancement [13].

The model compounds used in this study are analytes from the kynurenine pathway: L-tryptophan (L-TRP), kynurenine (KYN), 3-hydroxykynurenine (3-HK), kynurenic acid (KYNA), and two additional AAs, L-phenylalanine (L-PHE) and L-tyrosine (L-TYR). L-TRP is an essential AA for human functioning, but less than 1% of dietary tryptophan is directly used for protein synthesis. The majority undergoes decarboxylation, oxidation, hydroxylation, and transamination [14]. Approximately 80–90% of tryptophan is metabolized into kynurenine through the kynurenine pathway. In the initial step of this pathway, L-TRP is oxidized by tryptophan dioxygenase (TDO) or indoleamine 2,3-dioxygenase (IDO) to N-formylkynurenine, which is subsequently converted to kynurenine (KYN) [15]. KYN typically exists at low, micromolar concentrations [16] and is further metabolized within the kynurenine pathway by kynurenine aminotransferases (KAT I–IV), leading to the production of kynurenic acid (KYNA) through the irreversible transamination of KYN [17]. In the brain, KYNA is produced by astrocytes [18] and is present at micromolar concentrations, while in cerebrospinal fluid (CSF), it is found at nanomolar concentrations [19]. KYN can also be acted upon by kynurenine hydroxylase to produce 3-hydroxykynurenine (3-HK). An alternative metabolic route for KYN is its conversion to anthranilic acid (AA) by kynureninase. AA can then be further converted to 3-hydroxyanthranilic acid (3HAA). During the metabolism of 3HAA, 2-amino-3-carboxymuconate-6-semialdehyde (ACMS) is formed, which can be processed by 2-amino-3-carboxymuconate-6-semialdehyde decarboxylase [20] to produce picolinic acid (PCA). Alternatively, ACMS may undergo spontaneous cyclization to form quinolinic acid (QUIN). The concentration of QUIN in the brain and CSF must be maintained at relatively low levels, as an increase to 100 nM has been shown to exert neurotoxic effects [21]. QUIN is an agonist of the N-methyl-D-aspartate receptor (NMDAR), and its presence is associated with various neurological disorders and inflammatory conditions [22].

The kynurenine pathway is a source of compounds with diverse biological properties, often exhibiting opposing effects. Disruption of the balance between kynurenine concentrations due to dysregulation of in vivo mechanisms can lead to various issues and is often linked to pathological conditions. Understanding the concentrations of kynurenines associated with different diseases, as well as the methods for their detection for diagnostic purposes, may enable the development of new strategies for treating various pathological conditions, including infections, chronic inflammatory conditions, cancers, and fertility disorders. Currently, the determination of compounds from the tryptophan group is performed using analytical methods based on high-performance liquid chromatography coupled with mass spectrometry (HPLC-MS). However, due to the cost of these analyses, it is necessary to propose an economical yet equally reliable method for their determination based on spectrophotometric detectors, which are available in most analytical laboratories.

This study undertakes, for the first time, the application of online preconcentration using PAEKI in conjunction with a BGE containing mixed micelles aimed at the determination of anionic endogenous compounds. Tryptophan metabolites were selected as model compounds for this investigation.

Moreover, UV electrophoretic determinations are typically conducted for a maximum of three metabolites at a time, which does not provide a comprehensive view of changes and hinders full diagnostics. Additionally, the developed electromigration technique has the potential to serve as a new diagnostic tool for biochemical assessment where the balance between individual L-tryptophan metabolic pathways is disrupted. The electrophoretic methods described so far, summarized in Table 1, often encountered limitations related to significantly high concentrations of analytes (at the μM level).

Moreover, in this study, the simultaneous determination of the kynurenine pathway metabolites and AAs containing chromophores with a molecular weight similar was performed, which may interfere with their reliable quantification. Due to the high resolution provided by the developed BGE based on mixed micelles, simultaneous separation of kynurenines and aromatic AAs was achieved.

## 2. Results and Discussion

### 2.1. Mixed Micelle System

**Figure 2:**
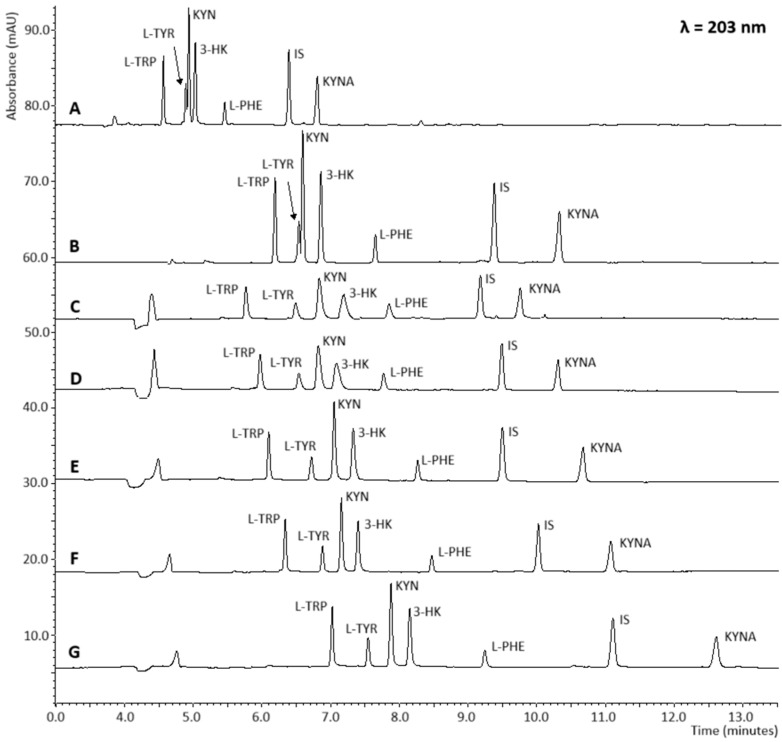
The electrophoretic separation of kynurenines at increasing concentrations of surfactants in the BGE, (**A**) without SDS and Brij-35, (**B**) 20 mM SDS, (**C**) 2 mM Brij-35, (**D**) 2 mM Brij-35 and 5 mM SDS, (**E**) 2 mM Brij-35 and 10 mM SDS, (**F**) 2 mM Brij-35 and 20 mM SDS, (**G**) 2 mM Brij-35 and 30 mM SDS. Other conditions: BGE: 20 mM Na_2_B_4_O_7_, 20% (*v*/*v*) MeOH; applied voltage 25 kV, HDI (5 s, 0.5 psi), capillary: 50 cm (length) × 50 µm. Analytical wavelength: 203 nm. Analyte concentration: each at 20 μg/mL. IS—internal standard (phenobarbital).

**Figure 3:**
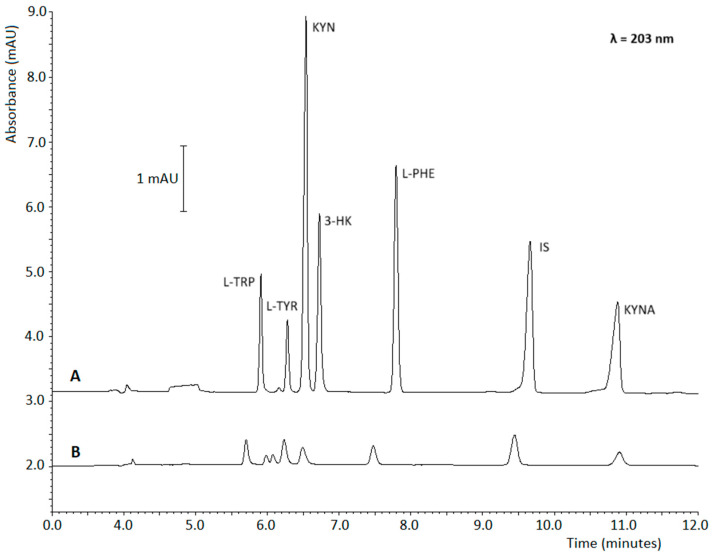
Signal enhancement effect for kynurenine pathway compounds using the PAEKI-MEKC technique and the mixed micelle system compared to standard HDI coupled with the MEKC separation. (**A**) PAEKI: 2 min, −6.5 kV, 0.5 psi, (**B**) HDI 5 s, 0.5 psi. Other conditions: BGE: 2 mM Brij-35, 20 mM SDS, 20 mM Na_2_B_4_O_7_, 20% (*v*/*v*) MeOH; applied voltage: 25 kV; capillary 50 cm (length) × 50 µm (I.D.). Analytical wavelength: 203 nm. Analyte and IS concentration: (**A**) 1 μg/mL, (**B**) 20 μg/mL.

Due to the minor differences in the molar masses of the analytes, the BGE for CZE did not allow for complete separation of the analytes, even at high concentrations of organic solvents (30% (*v*/*v*) MeOH). Therefore, the MEKC technique was chosen, which provides a high level of resolution in the electrophoretic separation system. Initially, a nonionic surfactant, Brij-35, which is a polymer of ethylene glycol, was employed. This allowed for the complete separation of all analytes in the range of 2–30 mM in BGE, with MeOH concentrations ranging from 20% to 30% (*v*/*v*); however, the peaks lost their sharpness. Brij-35 is a surfactant that increases the viscosity of aqueous solutions and bonds on the inner walls of the capillary, thereby inhibiting EOF and causing peak broadening [30]. Nevertheless, its undeniable advantage is its minimal impact on Joule heating. Similar separation results were obtained using BGE containing MeOH (20% (*v*/*v*)) and the anionic surfactant SDS at concentrations between 5 and 30 mM, where the peaks were completely resolved; however, the signal was broadened. Polar AAs such as L-TYR and L-PHE, through hydrogen bonding with water molecules, exhibit significant interactions with the hydrophilic (polar) head groups of SDS micelles. In contrast, nonpolar AAs and analytes with hydrophobic side chains, including KYN, 3-HK, L-TRP, and KYNA, primarily interact with the hydrophobic core of SDS as well as with neutral Brij-35 micelles [31]. Palmer et al. [32] observed that signals from analytes with low affinity for micelles broaden as the length of the injection capillary increases, which results from an expanded elution window. On the other hand, higher concentrations of SDS caused disturbances in the stacking process when using PAEKI, likely due to disruption of the counterion (cation) layer that stabilizes the stationary boundary formed between EOF and pressure during injection (Figure 2). A synergistic interaction between Brij-35 and SDS was employed as a separation mechanism. The addition of Brij-35 to SDS reduces electrostatic repulsion by diluting the anionic charges on the surface of SDS micelles. This diminishes repulsive forces between the polar headgroups of SDS, facilitating micelle formation and thereby lowering the critical micelle concentration (CMC). Conversely, incorporating SDS enables a reduction in the Brij-35 concentration required for complete analyte separation. This dual approach decreases the viscosity of the BGE and minimizes Brij-35 adhesion to the inner capillary wall, while maintaining optimal EOF stability. Figure 2 illustrates the separation of analytes at increasing concentrations of SDS in the BGE containing 2 mM Brij-35. As observed, at higher concentrations, the migration time of the analytes increased. Moreover, mixed micelle systems have been shown to sharpen peak signals during electrophoretic separations. Using PAEKI, the most effective separation for analytes was achieved with a BGE containing 20 mM SDS (Figure 2F), despite slightly higher peak intensities observed with the BGE containing 10 mM SDS (Figure 2E), which was associated with a minor shift in migration times (as can be seen in Figure 3). Ultimately, the final BGE composition consisted of 20 mM Na_2_B_4_O_7_, 20% (*v*/*v*) MeOH, 2 mM Brij-35, and 20 mM SDS.

### 2.2. PAEKI Parameters Optimization

The mechanism of PAEKI, as mentioned in the introduction, involves balancing the EOF using pressure, which is illustrated in Figure 1. The optimization of technical parameters for PAEKI is crucial, as it directly affects peak heights and the proper separation of all analytes. Therefore, it was essential to preliminarily develop the BGE composition, as the concentration ratio between Brij-35 and SDS must be carefully managed to achieve optimal EOF and peak resolution. For instance, while Brij-35 can stabilize EOF by reducing conductivity, its excessive use may hinder separation efficiency due to increased viscosity. Conversely, SDS enhances conductivity but can lead to increased EOF if not properly controlled. The investigated values for injection at pressure in PAEKI ranged from 0.3 to 1.0 psi (2.07–6.89 kPa). All separations were conducted using standard solutions of compounds of interest at a concentration of 1 µg/mL for each analyte, supplemented with phenobarbital sodium at a concentration of 1 or 0.25 µg/mL as an IS. For the optimization of pressure, the following values were maintained for other variables: applied voltage during injection at −6 kV, injection time at 1 min, capillary length of 50 cm with an inner diameter of 50 µm, and the BGE composed with 20 mM Na_2_B_4_O, 2 mM Brij-35, 20 mM SDS, and 20% (*v*/*v*) MeOH. Based on the results, it was concluded that the optimal pressure for the kynurenine samples is 0.5 psi, as this setting produced high, well-resolved, and sharp peaks. Higher pressures resulted in peak overlap, and a lack of peak symmetry, while lower pressures led to losses in the heights of the first five peaks.

The investigated injection voltage values ranged from −5.0 to −10.0 kV. The remaining parameters were consistent with those used in pressure optimization. Based on the results obtained from these analyses, it was determined that the most favourable voltage for the studied analytes is −6.5 kV—at this value, peaks were highest, while both higher and lower voltages resulted in diminished peak heights. For injection time optimization, the investigated values ranged from 1.0 to 3.0 min, while maintaining consistent parameters: pressure at 0.5 psi and voltage at 6.5 kV. An injection time of 2 min resulted in sharper and taller peaks compared to other configurations; however, injection times longer than 2 min caused peak interference.

Conclusively, the parameters for PAEKI preconcentration were established as follows: 0.5 psi, −6.5 kV, and an injection time of 2 min. Figure 3 shows signal enhancement with PAEKI compared to typical HDI.

Due to the different sample injection mechanisms (HDI and PAEKI), the peaks observed on the electropherograms may not exhibit identical migration times. Compared to HDI, both the pressure and voltage applied during PAEKI are subject to slight deviations from the target parameters programmed in the CE system and injection interface, for the same sample. As a result, it may take up to several seconds to establish the equilibrium between the EOF and the applied pressure. This relatively short stabilization period can affect the position of the sample injection zone, further contributing to differences in the migration times of analytes.

### 2.3. SPE Parameters Optimization

**Figure 4:**
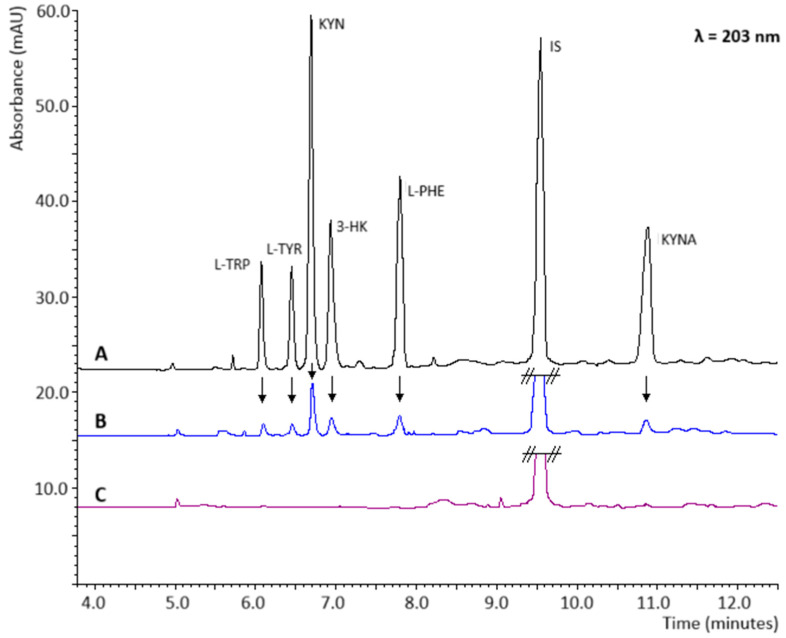
Representative electropherograms for random 5 mL artificial urine samples spiked with (**A**) 50 ng/mL each of analyte, (**B**) 5 ng/mL each of analyte, and (**C**) artificial blank urine samples. All samples were spiked with 100 ng/mL of the IS. Other conditions are the same as in Figure 3A.

Human urine collected from a healthy volunteer was initially selected as the biological material for investigation. Until analysis, the urine samples were stored at 4 °C. To ensure better binding to the column matrix, the following reagents were tested: 0.1 M HCl solution and 3% trichloroacetic acid (TCA) solution in acetonitrile (ACN), in order to fully protonate carboxyl groups (–COOH) and ionize amino groups (–NH_2_). Satisfactory results were obtained from trials conducted using 0.1 M HCl. Initially, an acid-to-sample ratio of 1:10 was employed; however, in subsequent experiments, the amount of HCl was reduced to 1:20. This reduction in HCl concentration did not influence analyte degradation. Although the analytes exhibited a strongly hydrophilic character, they differed in their solubility in water, log *p* values, and pKa values. The diversity of the physicochemical properties of the studied compounds necessitated testing various SPE columns, specifically hydrophilic–lipophilic Balance (HLB), hydrophilic cation exchange (HCN), and strong cation exchange (SCX) columns. The best results were obtained using SCX columns; tests on aqueous samples and artificial urine yielded the highest analyte peaks. Figure 4 illustrates the extraction of kynurenine from artificial urine spiked with standards and blank samples using SCX columns.

All compounds demonstrated thermolability, which required maintaining strict temperature conditions during sample preparation for analysis. For example, the evaporation of samples containing analytes took significantly longer—45 min—due to the need to use a lower temperature of 30 °C to prevent chemical degradation. The extraction procedure is detailed in Section 2.5.

### 2.4. Validation Study

The elaborated PAEKI-MEKC method has been validated according to the European Medicines Agency (EMA) guidelines for the validation of bioanalytical methods [33] by measuring peak heights at the analytical wavelength UV of maximum absorbance: 203 nm for all analytes.

#### 2.4.1. Linearity Range, LOD, and LOQ

The LOD and limit of quantification (LOQ) were calculated using the standard deviation (SD) derived from the maximum sensitivity provided by the electrophoretic system and were estimated by multiplying the SD of the blanks by factors of 3 and 10, respectively. Subsequently, the resulting LOD and LOQ values were validated separately by analysing six samples from different standards (*n* = 6) (Table 2). The linearity of the method was determined by adding analytes to an artificial urine matrix within the concentration ranges of 4–1000 ng/mL for KYN selected due to its lower LOD compared to other analytes, and 10–1000 ng/mL for others analytes, and 100 ng/mL for the IS with six replications (*n* = 6) at seven concentrations (10, 25, 50, 100, 250, 500, and 1000 ng/mL), for KYN with eight point (additionally at concentration 4 ng/mL). The relative peak heights were selected as analytical signals for all analytes and IS calibration curves were obtained through least squares linear regression analysis. Responses for kynurenines were linear within the studied concentration ranges (Table 2), which was confirmed by a satisfactory coefficient of determination (R^2^ ≥ 0.9987).

#### 2.4.2. Accuracy and Precision

The within-run accuracy and precision were calculated at the LLOQ (lower limit of quantification), LQC (low quality control), MQC (medium quality control), and HQC (high quality control) levels for five replicates, all from the same analytical run. For equivalent concentrations, accuracy and precision between runs were assessed over three different analytical cycles conducted on two different days. Based on the data presented in Table 3, it was confirmed that the values were within acceptable limits according to recommended guidelines.

#### 2.4.3. Dilution Integrity and Carry-Over

For dilution integrity, blank artificial urine samples were spiked with concentrations two times higher than the upper limit of quantification (ULOQ) (at 1 µg/mL for all analytes). These samples were then diluted with blank artificial urine (*n* = 6) to achieve concentrations of 400 and 200 ng/mL for all analytes. Diluted samples were assayed alongside the established calibration curve. The results of the dilution integrity investigation were within acceptable limits. The accuracy (%) and precision (% CV) were found to be in the ranges of 93.1 to 110.7 and 92.3 to 111.6, and precision 2.9–12.8, and 3.8–13.2, for within-run and between-run data, respectively, which is within the limit accepted by the guidelines, specifically ±15%. Carry-over effects were assessed by injecting three blank artificial urine samples into the capillary: one before and two after the injection of the ULOQ calibration standard. Consequently, no carry-over effects were observed.

#### 2.4.4. Stability

During the validation process, the stability of the analytes in stock solutions and urine samples was tested in triplicate (*n* = 3) for both LQC and HQC samples. All stability determinations were analysed against a freshly prepared calibration curve to obtain sample concentrations. The samples were subjected to short-term stability testing at room temperature for 6 h, post-preparative stability for 24 h at room temperature, and stability assessment after three cycles of freezing (−70 °C) and thawing. Long-term stability was evaluated at −70 °C, with stock solutions of analytes stored for 120 days at −20 °C and IS solutions stored for 30 days at 4 °C. Kynurenines in all samples stored at room temperature exhibited a decrease in concentrations, whereas stock solutions stored at refrigerated temperatures demonstrated reliable stability, as the mean of the test sample results was within the acceptance criteria of ≤15% of the initial control values. These results indicate that the analytes stored at temperatures of 4 °C or lower can be handled under normal laboratory conditions without significant losses during routine analysis.

### 2.5. Signal Amplification

This study addresses the limited sensitivity of UV detection in MEKC by optimizing an on-line preconcentration strategy utilizing the PAEKI technique. To quantify signal amplification in PAEKI-MEKC compared to conventional HDI, EFs were calculated as the ratio of LOD values between the two techniques. Evaluations were conducted under optimized PAEKI conditions and contrasted with the HDI protocol, where samples were injected hydrodynamically (5 s at 0.5 psi) using identical electrophoretic separation parameters. Without off-line preconcentration via SPE of urine samples, PAEKI-MEKC demonstrated a 46-fold signal increase for L-TYR and a 143-fold enhancement for KYN relative to standard HDI. Furthermore, the LODs achieved for kynurenines with PAEKI-MEKC were comparable to or lower than those reported in recent methodologies (Table 1).

### 2.6. Recovery

The absolute recoveries for kynurenine pathway metabolites and internal standard were determined at two different concentration levels (LQC, MQC, and HQC) with six replicates for each concentration by direct comparison of peak heights from extracted urine samples vs. non-extracted ones. Mean recoveries were in the range from 86.3 ± 5.9 to 93.7 ± 4.4% for LQC, 89.7 ± 4.7 to 95.1 ± 4.4% for MQC, and from 94.5 ± 5.4 to 100.2 ± 4.8% for HQC. The absolute recovery of I.S. (phenobarbital sodium) was 94.2 ± 3.9%. These data confirmed that the proposed extraction procedure for the metabolites from urine samples provided adequate sensitivity.

## 3. Materials and Methods

### 3.1. Chemicals and Reagents

L-TRP, L-KYN, 3-HK, KYNA, L-TYR, L-PHE, IS: phenobarbital sodium, SDS, Na_2_B_4_O_7_, HPLC-grade MeOH, as well as ACN were purchased from Sigma-Aldrich (St. Louis, MO, USA). Brij-35 and NaOH were purchased from Merck (Darmstadt, Germany). The deionized (DI) water (18.2 MΩ cm) used in all experiments was obtained from a Milli-Q apparatus from Milipore Sigma (Burlington, MA, USA). Phenobarbital sodium was selected as the IS due to its satisfactory water solubility, anionic form under separation conditions at pH > 9.0, and molecular weight similar to that of the analytes. Additionally, it does not interfere with their signals.

### 3.2. Urine Samples

In this study, the artificial urine solution was used for the estimation of validation parameters, dilution integrity, and carry-over; on the other hand, urine collected from healthy volunteers was used for the development of the extraction conditions, as well as stability. Informed consent was obtained from volunteers, and the study was approved by the Local Ethical Committee of the Medical University of Gdansk (no. NKBBN/366/2020). Due to the presence of the tested compounds in real human urine, all studies were conducted on artificial urine samples. Artificial urine solution was prepared by dissolving 3.034 g of urea, 0.042 g of uric acid, 2.0 of NaCl, 0.050 g of KCl, 0.285 g of NaH_2_PO_4_·2H_2_O, and 0.050 g of KH_2_PO_4_ (anhydrous) in 250 mL of deionized (18.2 M cm) water, and finally adjusted to pH 6.5 by 0.1 M NaOH solution. Urine samples were stored at a temperature of 4 °C until analysis.

### 3.3. Analyte Standard Solutions

Individual stock solutions of analytes at a concentration of 1 mg/mL were prepared in distilled water and stored at −20 °C in dark containers to prevent decomposition. Standard and working solutions (5, 0.25, 0.025 μg/mL) were freshly prepared during each working session from the stock solutions by appropriate dilution with 0.01 mM NaOH to ensure proper ionization of all analytes. All working solutions were stored at 4 °C in dark containers for up to 48 h.

### 3.4. Electrolyte and Surfactant Solutions

Stock solutions of SDS, Brij-35, and Na_2_B_4_O_7_ were prepared by dissolving solid substances in Milli-Q deionized water to final concentrations of 0.2 M, 0.1 M, and 0.1 M, respectively. These stock solutions were stored at 8 °C in a refrigerator for a maximum of two weeks. Working solutions and the BGE were freshly prepared daily by diluting the stock solutions with Milli-Q deionized water and were used within 24 h of preparation.

### 3.5. CE Instrumentation

A Beckman P/ACE MDQ system (Fullerton, CA, USA) equipped with DAD and a liquid-cooling device was employed. All experiments were performed in an uncoated fused-silica capillary (Polymicro Technologies, Phoenix, AZ, USA) of 50.2 cm (effective length 40 cm) with 50 µm i.d. The temperature of the separation was controlled at 25 (±0.1) °C by immersion of the capillary in cooling liquid circulating in the tube of the cartridge. The new capillary was conditioned with 0.1 M sodium hydroxide for 10 min, and deionized water for 2 min. The routine wash between runs was carried out every day using pressure with 0.1 M sodium hydroxide (1 min), deionized water (1 min), and rinse buffer (1 min) under positive pressure (at 50 psi = 344.7 kPa) applied at the injection end. Data processing was carried out using the 32 Karat System Software Version 5.0 P/N 715080 (Beckman Coulter).

### 3.6. Extraction Procedure

Artificial urine samples, as well as urine from healthy volunteers, were spiked with a mixture of analyte standards at concentrations of 1000, 500, 250, 100, 50, 25, 10 ng/mL, and additionally for KYN 4 ng/mL, along with an IS at a concentration of 100 ng/mL. SPE was performed using columns Supel-Select SPE SCX column (sulfonic acid functionalized hydrophilic modified styrene) from Sigma-Aldrich (Bellefonte, PA, USA), which were activated with 1 mL of MeOH followed by 1 mL of distilled water. To the 5 mL of spiked urine samples, 250 μL of 0.1 M HCl was added; subsequently, the mixture was centrifuged, and the supernatant was applied to the columns and washed with a 0.2% solution of formic acid in MeOH. After complete drying of the columns, the proper elution of the analytes was carried out using 1 mL of a 6% ammonia solution in MeOH. The obtained eluates were evaporated at a temperature of 40 °C and the samples were reconstituted using 100 µL of 0.01 mM NaOH solution, vortexed for 1 min, and cooled for at least 30 min in a refrigerator at 4 °C immediately prior to electrophoretic analysis.

## 4. Conclusions

This study represents the first attempt to enhance the applicability of PAEKI in combination with mixed micelles (SDS and Brij-35) as a pseudostationary phase for the analysis of anions and biogenic compounds in complex biological matrices, specifically human urine samples. The innovative application of PAEKI in conjunction with MEKC significantly increases the signal intensity of kynurenine pathway metabolites, achieving up to a 143-fold enhancement compared to the traditional HDI method. The developed PAEKI-MEKC method combined with SPE enables low detection limits for kynurenine (1.2 ng/mL) and other analytes (1.5–3.0 ng/mL), making it suitable for the simultaneous determination of multiple kynurenine pathway metabolites and AAs in biological samples, and thus useful for diagnostic applications. Dysregulation of the kynurenine pathway has been implicated in the pathogenesis of various diseases, including coronary artery disease. Moreover, this advancement not only solves the problems of existing techniques that often struggle with low sensitivity and specificity in the detection of anionic compounds and AAs but also provides a cost-effective alternative to the HPLC-MS/MS technique. Overall, this research contributes to the field of analytical chemistry by providing a robust methodology for the detection of biologically relevant anions.

## Figures and Tables

**Table 1:** The collection of recent electromigration methods for selected kynurenine determination in biological samples.

Analytes	Electromigration Method	Detection	LOD	Separation Time [min]	Sample Matrix	Reference
L-TRP, KYN, 3-HK, KYNA, L-TYR, L-PHE	PAEKI-MEKC	UV	1.2–3.0 ng/mL (5.7–15.8 pM) *	13	artificial urine, human urine	this study
L-TRP, KYN	CZE	UV	0.4–0.15 µM	9	human plasma	[23]
L-TRP, KYN, 3-HK, KYNA, AA, XA	CZE	MS	31 to 1000 nM	26	human plasma	[24]
L-TRP, KYN, 3-HK, KYNA	CD-CZE	UV	0.3 µg/mL (1.3 nM) *	8	homogenized tissues of Drosophila melanogaster	[25]
KYNA	CZE	LIF	1 nM	1	rat brain	[26]
L-TRP, KYN, KYNA	CZE	MS	20–67 nM	5	human CSF	[27]
D, L-KYN	Acid-mediated stacking-CD-CZE	UV	15 nM	10	human plasma and urine	[28]
L-TRP, KYN	LVSS-MEKC	UV	0.15–0.30 μM	17	human plasma	[29]

*—values converted to molar concentrations. AA—anthranilic acid, CD-CZE—cyclodextrin-capillary zone electrophoresis, CSF—cerebrospinal fluid, LVSS—large volume sample stacking, XA—xanthurenic acid.

**Table 2:** Statistical parameters for developed PAEKI-MEKC method with mixed micelle system. Linear range for analytes: 10–1000 ng/mL; only for KYN: 4–1000 ng/mL.

Analyte	PAEKI-MEKC (Mixed Micelle System)							HDI-MEKC (5 s, 0.5 psi)	EF Enhancement Factor
	Slope	Intercept	*R* ^2^	LOD [ng/mL]	LOQ [ng/mL]	Precision (%RSD)		LOD [ng/mL]	
						Migration Time	Peak Hight		
L-TRP	0.0023	−0.0231	0.9995	2.6	8.6	2.3	5.2	140	53.8
L-TYR	0.0016	−0.0039	0.9995	3	9.9	2.2	6.1	138	46.0
KYN	0.0042	0.0094	0.9996	1.2	4.0	1.9	7.9	172	143.3
3-HK	0.0027	−0.0398	0.9987	2.2	7.3	2.6	7.2	150	68.2
L-PHE	0.0030	−0.0139	0.9993	1.5	5.0	2.8	6.7	153	102.0
KYNA	0.0014	0.0006	0.9998	2.9	9.6	2.9	6.4	180	62.1

**Table 3:** The precision and accuracy data obtained from the elaborated method.

Analyte	Nominal Concentration [ng/mL]	Within-Run (*n* = 5)			Between-Run (*n* = 6)		
		Measured Concentration [ng/mL] (Mean ± SD)	Accuracy [%]	Precision [CV%]	Measured Concentration [ng/mL] (mean ± SD)	Accuracy [%]	Precision [CV%]
L-TRP	LLOQ 10	10.8 ± 1.3	108.1	11.4	10.9 ± 1.6	109.2	12.4
	LQC 25	23.0 ± 2.4	92.0	9.8	22.9 ± 2.7	91.6	11.5
	MQC 500	492.6 ± 23.5	98.4	7.0	485.6 ± 24.9	97.1	7.9
	HQC 1000	994.3 ± 32.6	99.4	4.1	992.7 ± 34.7	99.3	5.3
L-TYR	LLOQ 10	11.0 ± 1.4	109.7	11.0	11.1 ± 1.7	110.6	12.1
	LQC 25	23.4 ± 2.4	93.6	8.0	22.9 ± 2.8	91.6	8.3
	MQC 500	486.2 ± 24.2	97.2	3.9	484.8 ± 25.4	96.9	4.5
	HQC 1000	1012.4 ± 30.9	101.2	3.0	1017.4 ± 32.7	101.7	3.7
KYN	LLOQ 4	4.30 ± 0.4	107.5	11.9	4.5 ± 0.7	112.5	12.2
	LQC 25	25.8 ± 2.1	103.2	9.1	25.9 ± 2.4	103.6	9.5
	MQC 500	480.4 ± 17.6	96.1	5.9	477.4 ± 19.8	95.5	6.2
	HQC 1000	993.7 ± 30.1	99.4	5.0	989.6 ± 33.8	99.0	5.7
3-HK	LLOQ 10	11.0 ± 1.9	110.0	12.0	11.0 ± 2.1	110	12.2
	LQC 25	26.1 ± 2.4	104.4	9.9	26.2 ± 2.9	104.8	10.4
	MQC 500	476.0 ± 24.8	95.2	6.8	476.0 ± 26.4	95.2	7.4
	HQC 1000	1000.9 ± 34.9	100.1	3.0	1006.0 ± 36.9	100.6	3.3
L-PHE	LLOQ 10	10.8 ± 1.6	107.7	11.5	11.3 ± 1.9	112.7	12.8
	LQC 25	24.2 ± 2.5	96.8	10.0	24.0 ± 2.8	96.0	10.4
	MQC 500	484.5 ± 22.7	96.2	5.7	481.7 ± 24.4	96.4	6.1
	HQC 1000	1010.4 ± 35.7	101.0	4.8	1013.3 ± 37.7	101.3	5.1
KYNA	LLOQ 10	11.0 ± 1.7	109.9	10.1	11.2 ± 1.9	112.2	10.3
	LQC 25	26.4 ± 3.1	105.6	9.1	26.3 ± 3.3	105.2	9.7
	MQC 500	495.1 ± 22.8	99.0	6.0	490.3 ± 24.2	98.1	6.8
	HQC 1000	1011.1 ± 35.4	101.1	5.1	1023.0 ± 37.4	102.3	5.4

## Data Availability

The original contributions presented in this study are included in the article. Further inquiries can be directed to the corresponding author.
